# Prodigiosin-Producing *Serratia marcescens* as the Causal Agent of a Red Colour Defect in a Blue Cheese

**DOI:** 10.3390/foods12122388

**Published:** 2023-06-16

**Authors:** Javier Rodríguez, Cristina Lobato, Lucía Vázquez, Baltasar Mayo, Ana Belén Flórez

**Affiliations:** 1 Departamento de Microbiología y Bioquímica, Instituto de Productos Lácteos de Asturias (IPLA), Consejo Superior de Investigaciones Científicas (CSIC), Paseo Río Linares s/n, 33300 Villaviciosa, Spain; javier.rodriguez@ipla.csic.es (J.R.); cristina.lobato@ipla.csic.es (C.L.); lucia.vazquez@ipla.csic.es (L.V.); baltasar.mayo@ipla.csic.es (B.M.); 2 Instituto de Investigación Sanitaria del Principado de Asturias (ISPA), Avenida de Roma s/n, 33011 Oviedo, Spain

**Keywords:** *Serratia marcescens*, cheese, blue-veined cheese, technological defect, colour defect, traditional cheeses

## Abstract

Technological defects in the organoleptic characteristics of cheese (odour, colour, texture, and flavour) reduce quality and consumer acceptance. A red colour defect in Cabrales cheese (a traditional, blue-veined, Spanish cheese made from raw milk) occurs infrequently but can have a notable economic impact on family-owned, artisanal cheesemaking businesses. This work reports the culture-based determination of *Serratia marcescens* as the microbe involved in the appearance of red spots on the surface and nearby inner areas of such cheese. Sequencing and analysis of the genome of one *S. marcescens* isolate, RO1, revealed a cluster of 16 genes involved in the production of prodigiosin, a tripyrrole red pigment. HPLC analysis confirmed the presence of prodigiosin in methanol extracts of *S. marcescens* RO1 cultures. The same was also observed in extracts from red areas of affected cheeses. The strain showed low survival rates under acidic conditions but was not affected by concentrations of up to 5% NaCl (the usual value for blue cheese). The optimal conditions for prodigiosin production by *S. marscescens* RO1 on agar plates were 32 °C and aerobic conditions. Prodigiosin has been reported to possess antimicrobial activity, which agrees with the here-observed inhibitory effect of RO1 supernatants on different bacteria, the inhibition of *Enterobacteriaceae*, and the delayed development of *Penicillium roqueforti* during cheesemaking. The association between *S. marcescens* and the red colour defect was strengthened by recreating the fault in experimental cheeses inoculated with RO1. The data gathered in this study point towards the starting milk as the origin of this bacterium in cheese. These findings should help in the development of strategies that minimize the incidence of pigmenting *S. marcescens* in milk, the red defect the bacterium causes in cheese, and its associated economic losses.

## 1. Introduction

The qualitative and quantitative compositions of the microbiota of cheese contribute to determine the latter’s food safety, shelf-life, and sensorial properties [1]. Usually, the cheese microbiota involves a consortium of prokaryotic and eukaryotic microorganisms (and their viruses) that together have an organoleptic impact on ripened cheese via the degradation and transformation of milk constituents into flavour compounds [2]. Components of the microbiota may also influence the appearance of certain types of cheese via the development of fungi at the cheese surface (mouldy cheeses) or within the matrix (blue-veined cheeses) [3,4]. The appearance of cheese may be further influenced by the production of carotenoids that colour its rind (smear-ripened cheeses) [5]. Microorganisms have also been implicated in the production of excessive or atypical pigments causing colour defects that can negatively affect consumer acceptance and economic profitability [6,7,8]. The pigments involved in these defects, and the organisms that produce them, remain largely unknown. However, via the synthesis of indigo and indirubin, species of *Proteus* and *Psychrobacter* have recently been shown to produce a purple colour defect in a surface-ripened cheese [6]. Similarly, *Thermus thermophilus* strains that produce lycopene (a red carotenoid) have been associated with pink discolouration in a Continental-cheese type by recreating the defects using isolates from affected cheese [8]. Occasionally, discolouration is the result of the interaction between different cheese microbes. For instance, *Glutamicibacter arilaitensis* and *Penicillium* spp. acting together cause the overproduction of coproporphyrin III, leading to a pink marbling defect on the surface of some aged, smear-ripened cheeses [9].

Cabrales is a traditional, blue-veined cheese made from a mixture of raw cow, sheep, and goat milk; it is ripened in natural caves in the manufacturing area of northern Spain. It is protected via its European Protected Designation of Origin (PDO) status. The Cabrales microbiota involves a vast array of bacteria, yeast, and fugal species that succeed one another during manufacturing and ripening [10,11]. When cut, Cabrales cheeses are blue- or blue-grey-veined, the consequence of the profuse development of *Penicillium roqueforti* within the cheese matrix. Cabrales cheese is now usually made using acidifying and ripening starters based on mesophilic lactic acid bacteria and commercial *P. roqueforti* spores. However, due to the unpredictable microbial composition of raw milk and its associated microbial hazards, technological accidents still occur frequently with artisanal cheese manufacture [12]. During the 2021 winter–spring season, some ripened Cabrales cheeses with patent red spots in the cheese body (close to the surface) were detected at a number of cheesemaking facilities. This defect obliged the PDO Council to declare certain batches unsuitable for marketing. A pale-pink colouration was also observed in some curds and in 3-day old cheeses. Cabrales is produced by about twenty-two cheese makers following the operating instructions collected in its PDO technical annex at small family-owned facilities. There are no financial records by either cheese manufacturers or the PDO council of the cheeses affected by technical defects that cannot be put up for sale. The impact of the faults that occasionally occur, such as atypical reddish pigmentation, has not drastically influenced the family economy, at least so far; however, it might ultimately harm consumer acceptance.

For this reason, this study aimed to isolate, identify, and characterize the microorganism involved in the red colour defect that affects the quality of Cabrales cheese to obtain evidence for the design of strategies aimed at minimizing the impact on consumer confidence and profitability of cheeses. The results reported in this work include the following: (i) the isolation, identification, and characterization of a prodigiosin-producing strain of *Serratia marcescens* from a pigmented Cabrales cheese curd; (ii) the experimental recreation of the red discoloration in an experimentally manufactured cheese by inoculating cheese milk with the isolated bacterium. Thus far, the association of *S. marcescens* and red colour defects in food, specifically cheese, has not been previously reported in the literature. Therefore, these findings have the potential to lead to the development of strategies to improve cheese quality.

## 2. Materials and Methods

### 2.1. Microbial Sampling

One cheese curd (3-day old) and two pieces of ripened Cabrales cheese (>30-day-old), all with undesirable red patches on their surface and nearby inner matrix, were used in the identification of the microorganism responsible for this colour defect. Curd and cheese samples (from red areas) were used either directly, or diluted in sterile Ringer solution (Merck, Darmstadt, Germany), to inoculate plate count agar (PCA; Merck) supplemented with cheese (1%) and salt (3% NaCl) (PCACS), brain hearth infusion agar (BHI; VWR International, Radnor, PA, USA), 2xTY agar, and yeast glucose chloramphenicol agar (YGC; Merck) plates. All plates were incubated for 24 h up to one week at temperatures of 12–32 °C in order to recover the greatest possible bacterial and fungal diversity. Colonies with a reddish appearance were picked from plates and streaked on the same medium to obtain pure cultures.

### 2.2. Identification of Bacteria

Bacterial identification was performed by colony PCR using the primer pair 27 F (5′-AGAGTTTGATCCTGGCTCAG-3′) and 1492R (5′-GGTTACCTTGTTACGACTT-3′) to amplify part of the 16S rRNA gene. The PCR reaction mixtures, amplification conditions, amplicon purification, and sequencing process were as reported by Rodríguez et al. [13]. The DNA sequences obtained were compared against those in the NCBI database (https://www.ncbi.nlm.nih.gov/nuccore, accessed on 28 March 2022) using BLAST software 2.13.0 (https://blast.ncbi.nlm.nih.gov, accessed on 28 March 2022).

### 2.3. Genome Sequencing and Analysis

For genome sequencing, total DNA was extracted from an overnight culture using the QiAmp DNA Mini Kit (Qiagen, Düsseldorf, Germany). A standard genomic library of 0.5 kbp was constructed and paired-end sequenced (2 × 150 bp) at Eurofins Genomics (Ebersberg, Germany) using a NovaSeq 6000 System sequencer (Illumina, Inc., San Diego, CA, USA). Genome assembly and annotation were performed at the Bacterial and Viral Bioinformatics Resource Centre (BV-BRC; https://www.bv-brc.org/, accessed on 21 July 2022). For assembly, quality-filtered reads (Q > 30) were assembled in contigs using Unicycler software (https://github.com/rrwick/Unicycler, accessed on 21 July 2022). The genome sequence was examined for the presence of antimicrobial resistance and virulence genes by comparison against ResFinder (https://cge.cbs.dtu.dk/services/ResFinder/, accessed on 19 September 2022), CARD (https://card.mcmaster.ca/, accessed on 28 September 2022), NDARO (https://www.ncbi.nlm.nih.gov/pathogens/antimicrobial-resistance/, accessed on 3 October 2022), VFDB (http://www.mgc.ac.cn/VFs/, accessed on 7 October 2022) and Victors (http://www.phidias.us/victors/, accessed on 14 October 2022) databases. In addition, secondary metabolite biosynthetic gene clusters were sought using the AntiSMASH (http://antismash.secondarymetabolites.org, accessed on 14 November 2022) and BAGEL4 (http://bagel4.molgenrug.nl/ accessed on 21 November 2022) web servers. Other genome characteristics were manually examined. The genome sequence data of the detected *S. marcescens* RO1 was deposited in the GenBank database under the BioProject, Biosample and accession numbers PRJNA975739, SAMN35344818 and JASKOV000000000, respectively.

### 2.4. HPLC Analysis of the Red Pigment 

Pigment was extracted with 95% methanol from cells in plate cultures of the detected *S. marcescens* RO1, and from the red area of cheeses. Briefly, *S. marcescens* RO1 was plated on 2xTY agar and incubated at 32 °C for 24 h. Several colonies of this culture were suspended in 1 mL 95% methanol, and the suspension vortexed and then centrifuged at 13,000 rpm for 5 min. The supernatant was filtered, and the filtrate examined by reversed-phase high performance liquid chromatography (HPLC) using a Waters 2795 device (Waters, Milford, MA, USA). Cheese and curd samples (5 g) from red areas were homogenized in 95% methanol using an ultra-Turrax device, and the suspension was centrifuged for 5 min. The pellet was discarded, and the solvent quickly evaporated at 30 °C in a nitrogen flow. The dry extracts were then suspended in 1 mL 95% methanol and examined by HPLC. Two independent methanol extractions from supernatants of RO1 cultures, and curd and cheese samples were performed.

For all HPLC analyses, 10 µL methanol extract were analysed using a 5 μm Ascentis^®^ Express C18 column (Waters), a mobile phase gradient of water:acetonitrile:methanol (w:a:m) in 0.2% acetic acid, a flow rate of 1 mL/min, and an elution period of 15 min. The mobile phase gradient comprised w:a:m concentrations of 60:10:30 from min 1.0 to 4.0, of 10:10:80 from min 4.0 to 9.0, and of 0:20:80 from min 9.0 to 14.0. Compounds were identified using a 996 Photodiode Array Detector (Waters) at 535 nm.

### 2.5. Optimal Conditions for Prodigiosin Production

The effect of temperature, oxygen, and light intensity on the production of the red pigment by *S. marcescens* RO1 was assessed. For this, single colonies were picked and spread on PCACS and 2xTY plates, and incubated at different combinations of temperature (12, 22, 32, and 37 °C), atmosphere (aerobic and anaerobic), and light (in darkness and light) conditions. The assay was carried out in duplicate.

### 2.6. Prodigiosin Activity and Survival Conditions of Producing Strain

The antimicrobial effect of prodigiosin against bacterial species from the dairy environment was examined in agar-well diffusion tests. A prodigiosin solution was obtained by centrifugation and filtration (through a 0.2 µm membrane) of a suspension of the identified *S. marcescens* RO1 cells in sterile PBS solution previously grown on 2xTY plates at 32 °C for 24 h. A 30 µL volume of this solution was added to wells prepared in agar plates inoculated with the following indicator strains: *Lactococcus lactis* LMG6890^T^ (GM17), *Lactococcus cremoris* LMG6897^T^ (GM17), *Enterococcus faecalis* CECT481^T^ (GM17), *Streptococcus thermophilus* LMG6896^T^ (LM17), *Lactiplantibacillus plantarum* LMG6907^T^ (MRS), *Lacticaseibacillus casei* LMG6904^T^ (MRS), *Latilactobacillus sakei* CECT906^T^ (MRS), *Staphylococcus equorum* 16A1C (TSA), *Staphylococcus aureus* RN4220 (TSA), *Enterobacter* sp. Ent79 (2xTY), *Escherichia coli* DH10B (2xTY), *Debaryomyces hansenii* 1AD6 (YGC), *Kluyveromyces lactis* 3AD14 (YGC), *Penicillium roqueforti* PB6 (YGC), or *Geotrichum candidum* 3AM10 (YGC). For these experiments, 2xTY and TSA agars were made in-house from their components; all other media were from Merck (Darmstadt, Germany). Additionally, a well containing 30 µl of a sterile PBS solution was also included as control. Plates were incubated at the optimum temperature for each microbial indicator, and inhibition halos detected visually. Two replicates were independently evaluated.

The tolerance of *S. marcescens* RO1 to the conditions occurring during cheese manufacture, and ripening was examined by keeping the strain in PBS buffer supplemented with NaCl (1 up to 5%) under four different pHs (3.75–6). A cell suspension (McFarland 1) was used in inoculations for each set of conditions (≈10^6^ cfu/mL). The cells were maintained at room temperature for 6 days and the viability of RO1 was evaluated by plate counting on 2xTY. Three biological replicates for each condition were tested. Student’s *t*- test (paired *t*-test) was performed to compare the means between the two sampled variables.

### 2.7. Experimental Cheese Manufacture and Analysis

Two experimental batches consisting of control and RO1-inoculated cheeses were produced using raw cow’s milk following a blue-cheese manufacturing protocol. To mimic traditional Cabrales manufacture, no lactic acid bacteria (LAB) starter culture was used. Raw milk (20 L) was warmed at 32 °C and then rennet (1 × 10.000) and *P. roqueforti* spores (10^3^ cfu/mL) added. An overnight *S. marcescens* RO1 culture in milk (at 37 °C with shaking) was used to inoculate (1%) the RO1-inoculated batch. The curd was cut into hazelnut-size grains and, after whey drainage, placed in cylindrical moulds at room temperature (≈21 °C) without pressing. At 24 h the cheeses were unmoulded, covered with coarse salt, and turned over every day for 5 days. They were then placed in a ripening chamber at a controlled 12 °C and 80% humidity for 30 days.

To enumerate bacterial groups, samples of milk, curd, and cheese were aseptically removed and homogenized in a sterile 2% sodium citrate solution in a Colworth Stomacher 400 (Seward Ltd., London, UK) to obtain a 1:10 dilution. Ten-fold dilutions were then prepared in Ringer’s solution (Merck) and plated. Total aerobic mesophilic bacteria, lactococci, and lactobacilli were counted on PCA, GM17, and MRS plates, respectively, after incubation at 48 h at 32 °C. Enterococci were enumerated on Slanetz and Bartley (SB) plates (Merck) after incubation at 42 °C for 48 h. Staphylococci and Enterobacteriaceae were counted, respectively, on Baird Parker (BP) agar (Merck) supplemented with egg yolk tellurite solution (Merck), and on violet red bile dextrose (VRBD) plates (Merck), after incubation at 37 °C for 24 h. Finally, yeast and moulds were enumerated on YGC plates incubated at 25 °C for 3–5 days. The red coloration on the cheese surfaces was visually examined.

## 3. Results

### 3.1. Bacterial Association with the Red Colour Defect in Cheese

In an attempt to associate the red colour defect with a microorganism, media allowing the growth of a broad range of bacterial (BHI and 2xTY) and fungal (YGC) species were used, as was a medium mimicking cheese nutritive conditions (PCACS). White, beige and orange colonies of different sizes and brightness were abundant on plates of the different media inoculated with samples (or their dilutions) from the red area of two >30- day-old cheeses. After one week of growth, only cream-coloured yeast colonies and greenish *P. roqueforti*-like moulds were observed on the PCACS and YGC plates. No bacterial or fungal colonies with a colour compatible with the cheese defect were detected. The same results were obtained for the curd samples on BHI and 2xTY media. However, a brilliant red spot appeared on top of the bacterial growth lawn on one of the PCACS agar plates incubated at 32 °C. Pure red colonies were obtained after streaking on the same medium. One of the colonies was further purified, grown overnight in BHI, and stored at −80 °C; it was named RO1. Amplification, sequencing, and sequence comparison at the NCBI database, of a large segment of its 16S rRNA gene, identified RO1 as *S. marcescens* (>99% nucleotide identity). No red yeast or moulds colonies were detected for plated curd dilutions.

*S. marcescens* RO1 was subjected to whole genome sequencing and analysis. Using Unicycler software, a total of 8,153,858 high quality reads were assembled into 65 contigs, giving a genome size of 5,345,289 bp with an average G+C content of 59.41% (Table S1). Comparison of the RO1 genome against that of the type strain of *S. marcescens* (ATCC 13880^T^) gave dDDH and orthoANI values of 90.0% and 98.8%, respectively, confirming RO1 as belonging to *S. marcescens*. Functional analysis of the RO1 genome identified 5294 open reading frames (ORFs), of which 4289 had a functional assignment, 78 encoded tRNA molecules, and 4 encoded rRNA operons. Numerous genes involved in virulence (24) and antibiotic resistance (ampicillin, aminoglycosides, penicillin, tetracycline, and fosfomycin) were detected. Various genes encoding transposases and integrases involved in DNA mobilization were also identified, but no plasmid-associated sequences were found.

Several gene clusters coding for secondary metabolites with antimicrobial activity were detected across the RO1 chromosome. Notable clusters for the synthesis of bacteriocins (such as klebicin, bacteriocin_28b, microcin_H47, and vulnibactin) and antibiotics, including botromyicn, lankacidin C, and prodigiosin, were identified. Prodigiosin belongs to the prodiginine family, a group of red-coloured bacterial pigments. The putative operon for prodigiosin synthesis involved 16 genes (Figure S1), including *pigB*, *pigD,* and *pigE* which code for proteins involved in 2-methyl-3-n-amyl-pyrrole (MAP) synthesis, *pigA* and *pigF*-*pigN* which code for proteins involved in 4-methoxy-2,2′-bipyrrole-5-carbaldehyde (MBC) synthesis, and *pigC* which codes for a protein that condenses MAP and MBC to form prodigiosin. The structure of the gene cluster and its components in RO1 were identical to those in *S. marcescens* ATCC 274 [14].

### 3.2. Prodigiosin Production by S. marcescens RO1

**Figure 1:**
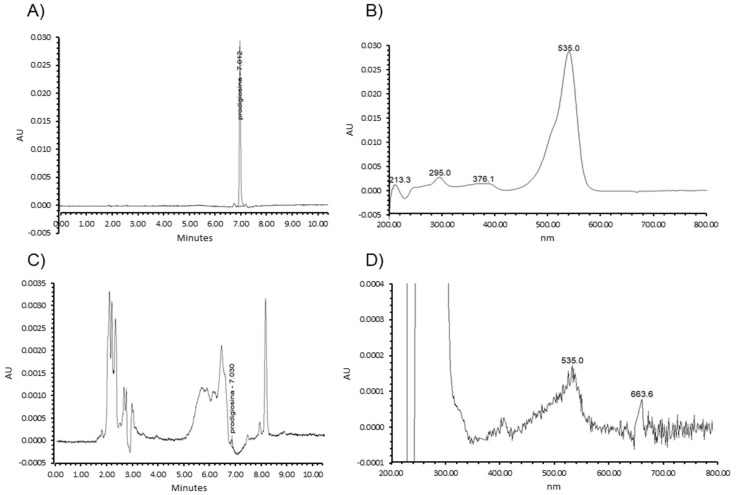
Detection and identification of the reddish pigment of Cabrales cheese using HPLC analysis. (**A**,**B**), chromatogram and absorbance profile of the crude extract obtained from a *S. marcescens* RO1 culture; (**C**,**D**), chromatogram and absorbance profile of the crude extract obtained from cheese with a reddish-coloured defect.

The production of prodigiosin by RO1, and the presence of this compound in cheese samples with red colour defects, was confirmed by HPLC analysis. The chromatographic profile of the red pigment from extracts of *S. marcescens* RO1 cells revealed a single peak at a wavelength of 535 nm, with a retention time of 7.012 min (Figure 1A,B).

For the methanol extracts of coloured areas of Cabrales cheeses, several peaks were observed at 535 nm (Figure 1C,D). Among those, a small peak with a retention time of 7.030 min compatible with that observed for *S. marcescens* RO1 was noted (Figure 1C). This peak, and that of the RO1 cells, showed an identical absorbance spectrum within the range 200–800 nm (Figure 1B,D), with the exception of a peak with a retention time of 663.6 min which is part of the eluent noise but only visible in cheese extracts due to the larger zoom of the spectrum applied. The chromatographic and absorbance profiles for prodigiosin agreed with those previously reported [15,16,17]; the small variations seen could have resulted from the use of different elution gradients.

**Figure 2:**
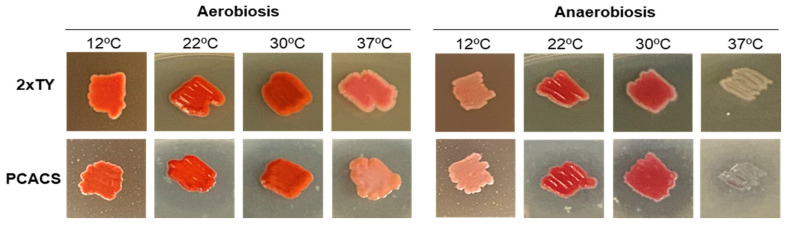
Morphology of *S. marcescens* RO1 colonies cultured on agar plates of 2xTY (up raw) and PCACS (bottom raw) media in aerobiosis (**left**) and anaerobiosis (**right**) and at temperatures ranging from 12 to 37 °C.

The requirement of nutrients related to the cheese environment by RO1 was assessed by comparing the growth of this strain on 2xTY and PCACS. No visual differences in the growth of *S. marcescens* RO1 on 2xTY and PCACS, nor in the production of the red pigment, were observed under any conditions (Figure 2). Although the strain grew well in anaerobiosis, a reduction in the red colour of the colonies was observed, particularly at 37 °C. Anaerobically-grown colonies developed the same red intensity as those grown in aerobiosis following their exposure to air for several hours. Incubation at 37 °C in aerobiosis was optimal for *S. marcescens* RO1 growth, while the production of prodigiosin was higher at lower temperatures (22–32 °C), especially in PCACS (Figure 2).

Since prodigiosin has been reported to show antimicrobial activity, the antimicrobial potential of the extract from *S. marcescens* RO1 was assessed by well-diffusion tests against common microbial biotypes found in cheese. The growth of most strains, which included Gram-negative and Gram-positive bacteria, yeasts, and moulds, was not significantly affected. However, small inhibition halos were seen around the wells containing the centrifuged and membrane-filtered RO1 suspension using *S. aureus* RN4220, *L. plantarum* LMG6907, *L. casei* LMG6904, and *Lc. lactis* LMG6890 (Figure S2) cultivated in deep semisolid media as test microorganisms.

### 3.3. Impact of pH and NaCl on RO1 Survival

**Figure 3:**
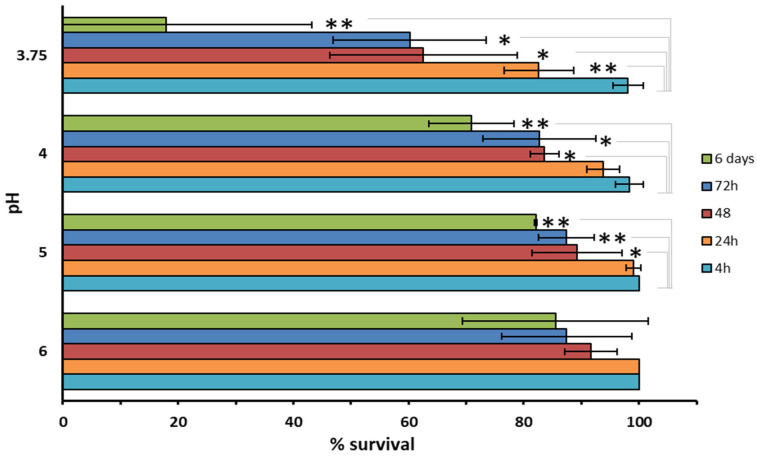
Survival (%) of *S. marcescens* RO1 over time in PBS buffer adjusted at different pH values. The data represent the mean ± standard deviation (SD) of three independent experiments. Asterisks indicate significant differences between the survival of RO1 at 4 h and longer exposure times (Student’s *t*-test, ** *p* < 0.01, * *p* < 0.05).

This assay was aimed at determining the capacity of *S. marcescens* RO1 to survive under the harsh conditions of cheesemaking. The acid tolerance of *S. marcescens* RO1 was variable depending on the pH and duration of exposure. Viability was maintained under all pH conditions after up to 4 h exposure (Figure 3) but decreased as the time of incubation increased from 4 h to 6 days (the lower the pH the stronger was this reduction). The differences on survival percentage were statistically significant between the exposure time of 6 days and 24 h or less in the pH range of 3.75 to 5. Nonetheless, RO1 proved to be quite resistant under low pH conditions, showing a viability of about 18% after 6 days at pH 3.75. No changes in viability were observed when the strain was grown in liquid medium supplemented with up to 5% salt (data not shown).

### 3.4. Recreation of the Red Colour Defect in Experimental Cheeses

**Figure 4:**
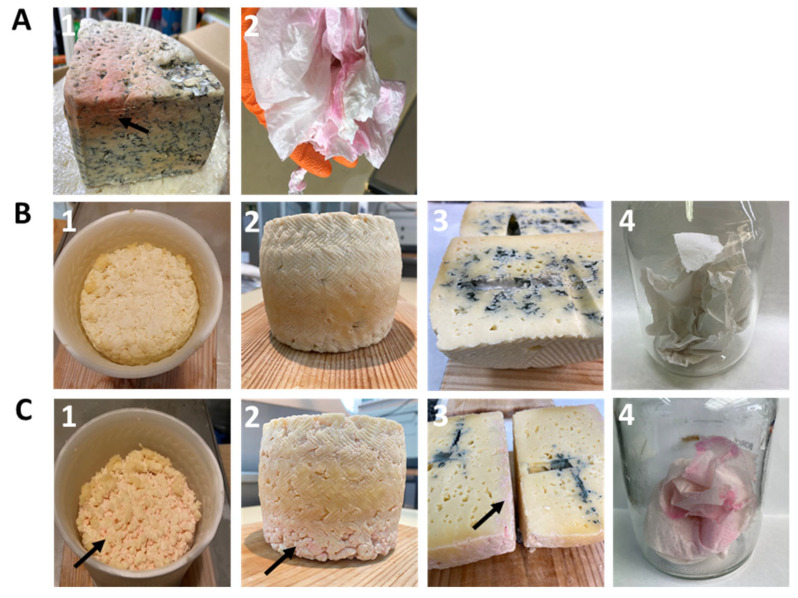
Experimental evaluation of the reddish-colour defect. Panel (**A**): Affected Cabrales cheese (**A1**) and a hand-drying paper supplied by cheesemakers (**A2**). Panels (**B**,**C**): Experimental blue-veined cheeses manufactured without (**B**) and with (**C**) the addition of *S. marcescens* RO1 (10^6^ cfu/mL final concentration) at different ripening stages (1–3) and hand-drying papers used from each batch (4).

To definitively associate *S. marcescens* with the red colour defect, two cheese batches—one inoculated with RO1 at 10^6^ cfu/mL, and one non-inoculated (control)—were manufactured at pilot-scale following a procedure resembling that for blue-veined cheese and without the addition of a LAB starter. Regular visual inspections of the cheeses throughout manufacturing and ripening (up to 1 month) revealed the development of pink/red coloration only on the cheese of the RO1-inoculated batch (Figure 4C).

A pink colour affecting the upper side of the curd surface and resembling that previously observed in the originally sampled Cabrales cheese curd was seen at 24 h during drying in moulds (Figure 4C(1)). The intensity of the colour defect in the original Cabrales cheese (Figure 4A(1)) was, however, not fully reached in the later stages of ripening (Figure 4C(2–3)). Anecdotally, papers used for hand-cleaning during the manufacture of the treated cheese (Figure 4C(4)) incubated under aseptic conditions at 32 °C showed pink/red stains; the same was reported by cheesemakers at the facilities where the original samples were collected (Figure 4A(2)).

Although the main purpose of experimental cheese manufacture was the evaluation of the development of the red colour defect by RO1 strain, microbiological analyses were also performed as a control of the microbial quality of the cheese during manufacturing and ripening. To this respect, *Enterobacteriaceae* were below the limit of detection (<100 cfu/g) in the control cheese on day 1, but reached 10^6^ cfu/mL in the treated cheeses (the intended inoculum size of *S. marcescens* RO1) (Table 1). Surprisingly, the number of *Enterobacteriaceae* increased significantly in the control cheese after day 1 and was maintained all along ripening. In contrast, none were detected in the treated batch after day 7. No differences in microbial counts were recorded for total mesophilic bacteria, lactococci, lactobacilli, enterococci, or micrococci-staphylococci between the inoculated and control cheeses. However, *P. roqueforti* development was clearly delayed in the inoculated cheeses (Table 1).

## 4. Discussion

A considerable percentage of cheeses produced in Europe are discarded due to the presence of pathogenic bacteria of mandatory declaration [18]. The impact of technological defects on the economy of the cheese industry is, however, largely unknown. Among these technological defects, undesirable colorations (red, pink, brown, purple, etc.), mostly affecting the rind, have long been reported for a variety of cheese types, especially smear-ripened cheeses [19]. Some strains (of both prokaryotic and eukaryotic organisms) have already been linked to the production of pigmented molecules responsible for defects in cheese [6,8,9,20]. The identification of the microorganisms responsible for colour defects is of the utmost importance since strategies aimed at eliminating or inhibiting their development, and thus minimizing economic losses, rely on this information.

The complex microbiota of farm-made Cabrales cheese, which includes a great diversity of prokaryotic and eukaryotic organisms [21], makes it difficult to identify spoilage biotypes, which might be present in smaller numbers than the majority populations. In the present work, culture-based analyses involving media combining different carbon sources, NaCl concentrations, and culture conditions (time and temperature), allowed the isolation-from a curd sample-of an *S. marcescens* strain that produced prodigiosin, a cell-associated red pigment [22]. *S. marcescens* is a near ubiquitous, rod-shaped Gram-negative bacterium of the family *Yersiniaceae* [23]. It is widespread in farm environments (water, bedding material, bulk tank milk, teat dip, etc.) [24] and some strains can cause opportunistic infections, including mastitis in both women and dairy cows [25,26]. A preliminary screening for *S. marcescens* in milk from our region using a chromogenic agar detected about 11% of all members of *Enterobacteriaceae* present to belong to this species. None of them, however, produced red colonies on BHI agar, whereas prodigiosin-producing *S. marcescens* strains were occasionally recovered from the cheese milk used in Cabrales manufacture. 

In addition to the isolation of *S. marcescens* RO1 from cheese curd, its production of the red colour defect was supported by the chromatographic analysis of methanol extracts from affected cheeses and RO1 cultures, which showed chromatograms and absorbance profiles compatible with prodigiosin [15,16,17]. Moreover, the sequence analysis of the RO1 genome showed (on its chromosome) the potential to produce this pigment. The RO1 pig gene cluster showed high nucleotide identity (≥99%) and an identical genetic structure to that of *S. marcescens* ATCC 274 [14].

Prodigiosin has recently received interest due to its antioxidant activity (which improves the immune system), its antimicrobial activity against bacterial, fungal and yeast species [15,27,28,29], and its antiproliferative effect on cancer cells [29,30,31]. Antimicrobial activity (although low level) against some bacterial strains was demonstrated in agar diffusion tests in the present study, as it was against other members of *Enterobacteriaceae* under experimental cheesemaking conditions. The delayed development of *P. roqueforti* in the RO1-inoculated batch may also be attributable to the prodigiosin content of the cheese. However, genome analysis of RO1 also revealed its potential to produce klebicin, bacteriocin-28b, microcin-H47, vulnibactin, pyrronazol, and other compounds reported to have antimicrobial activity, particularly against Gram-negative bacteria [15,32,33,34]. Either alone or in combination, these compounds may account for the antimicrobial activity of strain RO1.

Although the experimental manufacturing process did not reproduce traditional Cabrales cheese, the addition of *S. marcescens* RO1 to the raw milk confirmed its association with the red colour defect. Differences in cheese manufacturing, ripening conditions and/or interactions between components of the traditional cheese microbiota might have accounted for the colour differences seen between the defect in the experimental cheeses compared to conventional ones. The fact that *S. marcescens RO1* was not detected in the experimental treated cheeses beyond day 7 is consistent with the non-recovery of viable prodigiosin-producing cells in the ripened Cabrales cheeses with colour defects analysed in this study. In addition to the low pH (≈5.0 in Cabrales cheese at day 3 [11]), other LAB metabolites, such as organic acids, hydrogen peroxide, or bacteriocins [35], might also be inhibitory to *S. marcescens* RO1 in cheese. The need for prodigiosin-producing *S. marcescens* to attain high numbers (>10^6^ cfu/mL) to produce the red defect, and its susceptibility to manufacturing conditions, suggest the origin of this bacterium to be the cheese milk, and that it grows early during cheese manufacture.

## 5. Conclusions

This work reports a red colour defect in Cabrales cheese for the first time. A prodigiosin-producing strain of *S. marcescens* was isolated from a cheese curd and found to be the microorganism responsible for this defect. This is the first evidence of *S. marcescens* associated with a red colour defect in food, specifically a blue cheese. Although the source of *S. marcescens* needs to be investigated further, the requirement of a high level of bacterial cells for the red defect to develop in experimental cheeses and the occasional presence of prodigiosin-producing *S. marcescens* strains in the milk destined for Cabrales manufacture suggests that it indeed comes from the cheese milk. The prevention of clinical and subclinical mastitis caused by *S. marcescens* in cattle and keeping the time short between milk collection and cheese manufacture could help prevent *S. marcescens* replication to the levels required for cheese staining to occur.

## Figures and Tables

**Table 1:** Changes in counts (log10 cfu/g) of microbial groups of control and *S. marcescens* RO1-inoculated experimental, blue-veined cheese batches throughout manufacturing and ripening.

Microbial Group	Condition	Milk	Curd	Ripening Time (Days)			
				3	7	15	30
Total mesophilic bacteria	Control	4.79	6.05	8.96	8.88	8.05	8.78
	Inoculated		6.65	8.88	8.59	7.49	8.26
Lactococci	Control	3.61	5.53	8.99	8.87	7.96	8.80
	Inoculated		6.51	8.42	8.77	7.45	8.16
Lactobacilli	Control	3.13	5.76	8.71	7.45	6.56	7.27
	Inoculated		5.74	8.19	7.45	6.93	7.37
Enterococci	Control	3.06	4.71	8.78	8.20	8.05	8.58
	Inoculated		5.43	8.20	8.33	7.34	8.43
Micrococci and staphylococci	Control	3.46	5.24	8.37	7.59	7.83	7.89
	Inoculated		5.41	7.82	7.17	7.00	6.87
Enterobacteriaceae	Control	<2	<2	6.42	5.90	5.51	4.03
	Inoculated	6.59	5.65	4.30	<2	<2	<2
Yeasts and moulds	Control	2.68	<2	<2	3.78	6.07	7.66
	Inoculated		<2	<2	<2	<2	6.47

## Data Availability

The data used to support the findings of this study can be made available by the corresponding author upon request.

## Supplementary Materials

The following supporting information can be downloaded at: https://www.mdpi.com/article/10.3390/foods12122388/s1, Figure S1: Prodigiosin biosynthetic clusters (pig cluster) from *Serratia marcescens* RO1. Figure S2: Agar well diffusion test; Table S1: General features of the whole genome sequence of *S. marcescens* RO1 isolated from a pink-coloured curd.
