# Supplementary material for: Prodigiosin-Producing Serratia marcescens as the Causal Agent of a Red Colour Defect in a Blue Cheese
Source: Foods. 2023 Jun 16;12(12):2388. doi: 10.3390/foods12122388 (PMC10297559; doi:10.3390/foods12122388)
Supplement: Supplementary file 1 [file foods-12-02388-s001.zip › Supplementary Fig_1_cluster prodigiosin.pptx]

## Slide 1
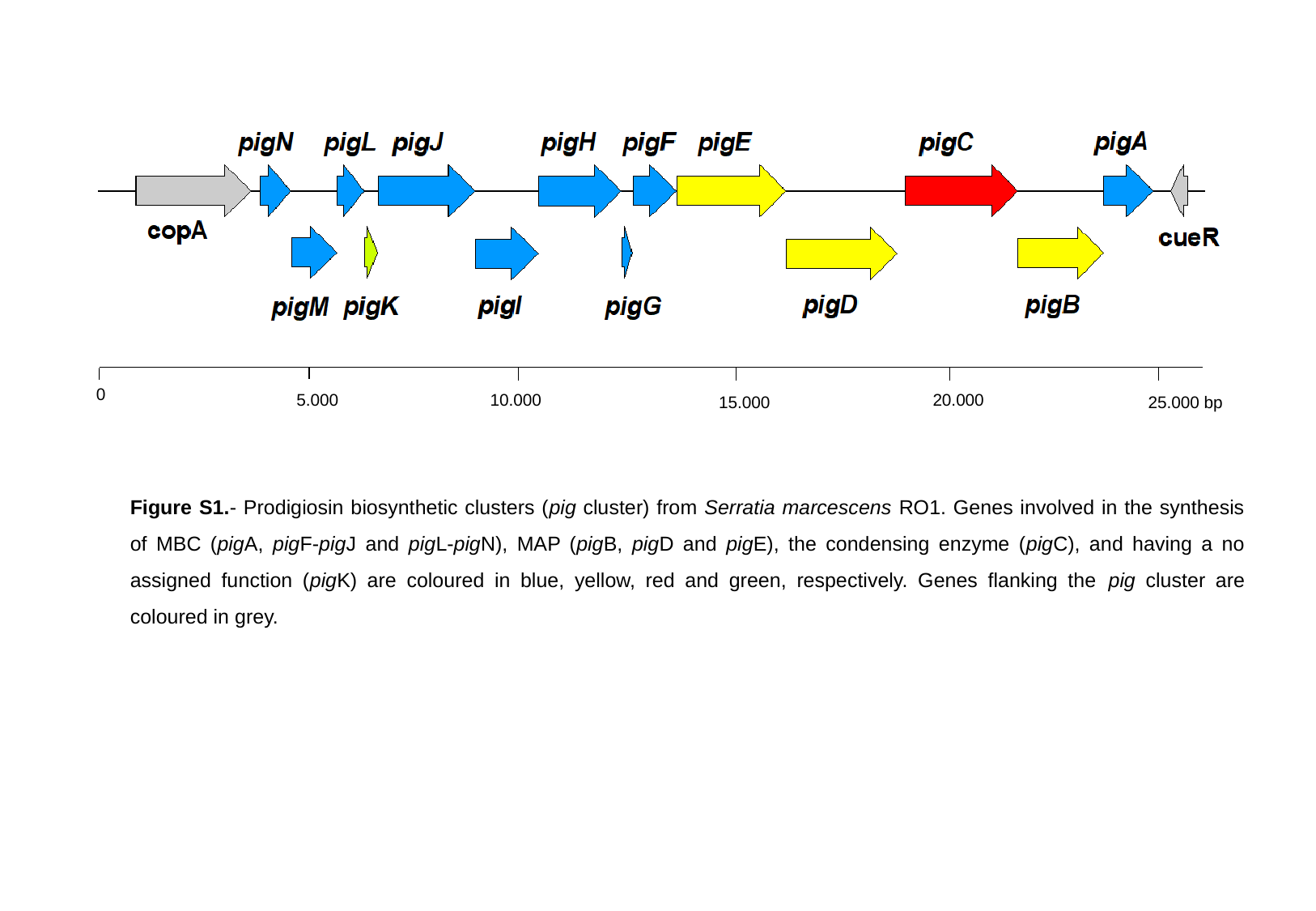

0
5.000
10.000
20.000
15.000
25.000 bp
Figure S1.- Prodigiosin biosynthetic clusters (pig cluster) from Serratia marcescens RO1. Genes involved in the synthesis of MBC (pigA, pigF-pigJ and pigL-pigN), MAP (pigB, pigD and pigE), the condensing enzyme (pigC), and having a no assigned function (pigK) are coloured in blue, yellow, red and green, respectively. Genes flanking the pig cluster are coloured in grey.
