# Supplementary material for: Prodigiosin-Producing Serratia marcescens as the Causal Agent of a Red Colour Defect in a Blue Cheese
Source: Foods. 2023 Jun 16;12(12):2388. doi: 10.3390/foods12122388 (PMC10297559; doi:10.3390/foods12122388)
Supplement: Supplementary file 1 [file foods-12-02388-s001.zip › Supplementary Fig_2_antimicrobial.pptx]

## Slide 1
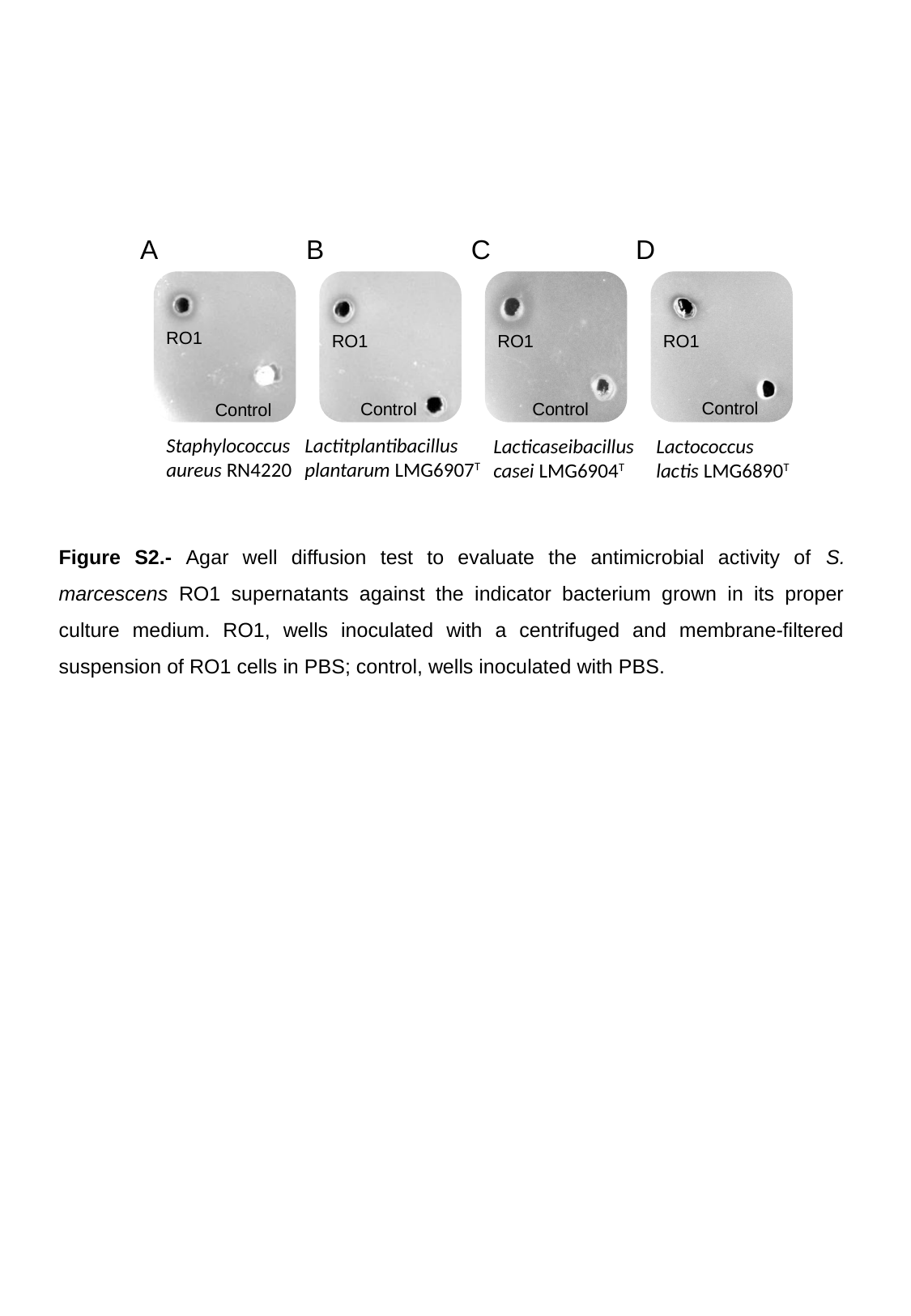

D
C
B
A
RO1
RO1
RO1
RO1
Control
Control
Control
Control
Staphylococcus aureus RN4220
Lactitplantibacillus plantarum LMG6907T
Lacticaseibacillus casei LMG6904T
Lactococcus lactis LMG6890T
Figure S2.- Agar well diffusion test to evaluate the antimicrobial activity of S. marcescens RO1 supernatants against the indicator bacterium grown in its proper culture medium. RO1, wells inoculated with a centrifuged and membrane-filtered suspension of RO1 cells in PBS; control, wells inoculated with PBS.
