# Supplementary material for: Prodigiosin-Producing Serratia marcescens as the Causal Agent of a Red Colour Defect in a Blue Cheese
Source: Foods. 2023 Jun 16;12(12):2388. doi: 10.3390/foods12122388 (PMC10297559; doi:10.3390/foods12122388)
Supplement: Supplementary file 1 [file foods-12-02388-s001.zip › Supplementary Table S1.pdf]

Table S1.- General features of the whole genome sequence of *S. marcescens* RO1 isolated from a pink-coloured curd.

**General genome features**	***S. marcescens* RO1**
Genome size (bp)	5.345.289
% G+C content	59.41
No. of contigs	65
No. of indicated protein-encoding genes	
Total	5294
With functional assignments	4289
Hypothetical	1005
No. of indicated RNA genes	
rRNA operons	4
tRNA molecules	78
Virulence Factors (VFDB)	24<br>Flagellum, Lipopolysaccharide
Antibiotic Resistance genes	5<br>*ampC, acc(6)-Ic, macAB, tetA, fosA*
Genes encoding plasmid replication proteins	0
Genes coding for transposases/integrases/excisionases	4/11/0
Pathways of secondary metabolites	8
